# Rapid detection of *Mycoplasma hyopneumoniae* by recombinase-aided amplification combined with the CRISPR/Cas12a system

**DOI:** 10.3389/fcimb.2024.1469558

**Published:** 2024-12-20

**Authors:** Kaili Li, Tingyu Luo, Yu Zhang, Changwen Li, Hongyan Chen, Changyou Xia, Caixia Gao

**Affiliations:** ^1^ State Key Laboratory for Animal Disease Control and Prevention, Harbin Veterinary Research Institute, Chinese Academy of Agricultural Sciences, Harbin, China; ^2^ Heilongjiang Provincial Key Laboratory of Laboratory Animal and Comparative Medicine, Harbin Veterinary Research Institute, Chinese Academy of Agricultural Sciences, Harbin, China; ^3^ National Poultry Laboratory Animal Resource Center, Harbin Veterinary Research Institute, Chinese Academy of Agricultural Sciences, Harbin, China

**Keywords:** *Mycoplasma hyopneumoniae*, CRISPR/Cas12a, recombinase-aided amplification, visualization, rapid detection

## Abstract

*Mycoplasma hyopneumoniae* (*M. hyopneumoniae*) is one of the primary agents involved in porcine respiratory disease complex, and circulates in the swine industry worldwide. The prevention and control of *M. hyopneumoniae* is complicated. Thus, a recombinase-aided amplification (RAA) assay coupled with the clustered regularly-interspaced short palindromic repeats (CRISPR)/Cas12a system was established for the detection of *M. hyopneumoniae*. The most suitable primer pairs and CRISPR RNA (crRNA) were screened and selected for the RAA-CRISPR/Cas12a detection system. We have achieved a detection limit of 1 copy/µL and 5 copies/µL per reaction for the RAA-CRISPR/Cas12a-fluorescence assay and RAA-CRISPR/Cas12a-lateral flow assay (LFA), respectively. Furthermore, the RAA-CRISPR/Cas12a system displayed no cross-reactivity with other respiratory pathogens. The performance of the RAA-CRISPR/Cas12a system was compared with PCR as recommended by the Chinese national standard (GB/T 35909-2018) and qPCR as recommended by the Chinese entry–exit inspection and quarantine industry standard (SN/T4104-2015) for clinical samples, and good consistency with these methods was observed. Above all, the methods shed a light on the convenient, portable, visual, highly sensitive and specific detection of *M. hyopneumoniae*, demonstrating a great application potential for on-site monitoring of *M. hyopneumoniae* in the field.

## 1 Introduction

*Mycoplasma hyopneumoniae* (*M. hyopneumoniae*) is considered one of the smallest known bacteria and lacks a cell wall, with a genome size of approximately 0.86–0.96 Mb, including the *P36* gene which has been shown to be highly homologous (Caron et al., 2000). The P36 gene also has high specificity for differentiating *M. hyopneumoniae* from other microorganisms (Caron et al., 2000). *M. hyopneumoniae* serves as the primary pathogen responsible for enzootic pneumonia in swine. Additionally, it plays a crucial role as one of the key agents in the Porcine Respiratory Disease Complex (Zimmer et al., 2020). *M. hyopneumoniae* is widespread worldwide and detection rates have risen dramatically since 2018, causing major economic losses to the swine industry (Maes et al., 2018; Zhang et al., 2021). The increase in prevalence can be ascribed to the heightened focus on control measures for African swine fever for its initial outbreak in China in 2018 (Assavacheep and Thanawongnuwech, 2022; Zhang et al., 2021). Furthermore, vaccination against *M. hyopneumoniae* is not compulsory and vaccines are not widely utilized on farms (Zhang et al., 2021). Antibiotic treatment (tetracyclines, macrolides and lincosamides) can alleviate clinical symptoms and reduce bacterial load in infected individuals, but do not eliminate the infection so that a risk of reinfection (Maes et al., 2008; Zhang et al., 2021). *M. hyopneumoniae*, which can reside in the tonsils and nasal cavities of healthy pigs, can also induce disease when isolated from apparently unaffected animals (Garza-Moreno et al., 2022). Therefore, rapid diagnosis is crucial to prevent and control an epidemic of *M. hyopneumoniae*. Currently, the main detection methods regarding *M. hyopneumoniae* in swine include isolation culture, serologic assays and nucleic acid assays. Although identification of *M. hyopneumoniae* through bacterial culture is regarded as the “gold standard”, isolation is difficult due to its fastidious growth requirements and slow growth rate (Dubosson et al., 2004; Thacker, 2004). Many serological assays for *M. hyopneumoniae* have been developed to monitor the health status of pig herds (Ding et al., 2021; Feng et al., 2014; Liu et al., 2016), but most serological assays cannot distinguish between natural infections and vaccinated animals, leading to potential false-positive results (Sibila et al., 2009). Nucleic acid assays for *M. hyopneumoniae*, such as polymerase chain reaction (PCR) (Cai et al., 2007; Canturri et al., 2024), nested PCR (nPCR) (Calsamiglia et al., 1999; Moiso et al., 2020) and real-time quantitative PCR (qPCR) (Dubosson et al., 2004; Strait et al., 2008), have been used for detection in the laboratory. Moreover, the PCR, nPCR and qPCR detection methods for *M. hyopneumoniae* were also adopted in the Chinese national standard (GB/T 35909-2018), Chinese agricultural industry standard (NY/T 1186-2017) and Chinese entry–exit inspection and quarantine industry standard (SN/T 4104-2015). However, relatively expensive instruments such as PCR machines and electrophoresis apparatus are necessary to perform these tests, which restrict their use in many small front-line laboratories. Techniques such as recombinase polymerase amplification (Liu et al., 2019), recombinase-aided amplification (RAA) (Li et al., 2023) and loop-mediated isothermal amplification (Li et al., 2013) have been developed as alternative approaches. Compared to traditional PCR technology, isothermal amplification does not need expensive experimental equipment and consumables, and therefore has a wider application range and easier operation. However loop-mediated isothermal amplification requires more than four specific primers and complicated primer design. In addition RAA and recombinase polymerase amplification are susceptible to non-specific amplification at low template concentrations or in the absence of a template because they operate under isothermal conditions.

In recent years, the CRISPR/Cas system, combined with the shorter CRISPR RNA (crRNA) and Cas proteins (Yuan et al., 2022), has emerged as a potential tool for rapid, sensitive and highly-specific molecular diagnosis (van Dongen et al., 2020), which relies on the cleavage preferences of Cas12 or Cas13 in a nonspecific way after binding to a specific target DNA or RNA through crRNA (Li et al., 2018a). Among those, the Cas12a-based system using a single-stranded DNA probe is more suitable for the detection of bacterial pathogens (Chen et al., 2018; Yao et al., 2018). The combination of the CRISPR/Cas system with isothermal amplification can be easily used for the detection of nucleic acids, such as SHERLOCK (Kellner et al., 2019), DETECTR (Chen et al., 2018) and HOLMES (Li et al., 2018b), and the result can be combined with fluorescent probes or immunochromatography technology to express results. CRISPR/Cas12a-based detection has been successfully applied to detect porcine respiratory bacterial pathogens, such as *Actinobacillus pleuropneumoniae* (*A. pleuropneumoniae*)*, Streptococcus suis* (*S. suis*)*, Haemophilus parasuis* (*H. parasuis*) and *Pasteurella multocida* (*P. multocida*) (Hao et al., 2023; Luan et al., 2022; Wang et al., 2024; Zhang et al., 2023).

The P36 gene of *M. hyopneumoniae* is suitable for designing primer and probe to enhance detection accuracy and sensitivity (Caron et al., 2000). It enables rapid and precise identification of *M. hyopneumoniae* in clinical samples for early diagnosis and treatment. The P36 gene of *M. hyopneumoniae* has previously been reported in PCR and loop-mediated isothermal amplification (LAMP) detection methods (Caron et al., 2000; Liu et al., 2015). In this study, primers for RAA and crRNA *in vitro* transcription templates based on the *P36* gene of *M. hyopneumoniae* were designed. A rapid detection platform for *M. hyopneumoniae* based on RAA with the CRISPR/Cas12a system was successfully developed, which was not only highly sensitive and specific, but even suitable for detection at front-line sites. It provides a highly-convenient method of microbial quality control of specific pathogen-free pigs and of field diagnosis and detection in the swine industry.

## 2 Materials and methods

### 2.1 Pathogenic nucleic acids and clinical samples

Genomic (DNA or cDNA) of *M. hyopneumoniae*, *Mycoplasma hyorhinis* (*M. hyorhinis*), *A. pleuropneumoniae*, *H. parasuis*, *S. suis*, *P. multocida*, porcine reproductive and respiratory syndrome virus (PRRSV), swine influenza virus (SIV), porcine circovirus type 2 (PCV2), pseudorabies virus (PRV), *Mycoplasma capricolum* (*M. capricolum*), *Mycoplasma synoviae* (*M. synoviae*) and *Mycoplasma gallisepticum* (*M. gallisepticum*) were stored in the State Key Laboratory for Animal Disease Control and Prevention, Harbin Veterinary Research Institute, Chinese Academy of Agricultural Sciences. In addition, we gratefully acknowledge the contribution of 51 lung tissue samples and 25 nasal swab samples from pigs, kindly provided by other laboratories at the Harbin Veterinary Research Institute. The Animal Ethics Committee of Harbin Veterinary Research Institute did not require ethical review or approval for this study.

### 2.2 Design and synthesis of crRNA

The *P36* gene of *M. hyopneumoniae* has been shown to be highly homologous (Caron et al., 2000), so we selected it as a test gene and used fragments of the conserved region for the design of crRNA. Four DNA templates for crRNA synthesis were designed using the CHOPCHOP online tool (Montague et al., 2014). The four DNA templates were synthesized by Sangon Biotech Co., Ltd. (Shanghai, China). The four DNA templates underwent an annealing heat treatment. Subsequently, *in vitro* transcription was performed by incubating at 37°C for 16 hours, following the instructions provided by the HiScribe T7 Quick High Yield RNA Synthesis kit (New England Biolabs, Beverly, MA, USA). The final transcription products were treated with DNase I (New England Biolabs) to remove residual DNA template and purified using the Monarch RNA Cleanup Kit Protocol Card (New England Biolabs). The concentration of the final crRNA was measured using a NanoDrop spectrophotometer (Thermo Fisher Scientific, Waltham, MA, USA) and stored at -80°C for future use. The sequences of all oligonucleotides synthesized in this study are presented in **Table 1**.

**Table 1:** Primers and probes used in this study.

Name	Sequences (5′-3′)	Reference
crRNA1	UAAUUUCUACUAAGUGUAGAUGCAAUAGCUGCACCAAUUCCAUAA	This study
crRNA2	UAAUUUCUACUAAGUGUAGAUUCCGUGAAAUCCGUAUUCUCCUCG	
crRNA3	UAAUUUCUACUAAGUGUAGAUAUAAGCCCGGCGAGAAACUGGAUA	
crRNA4	UAAUUUCUACUAAGUGUAGAUCUCCGUGAAAUCCGUAUUCUCCUC	
RAA-F1	ACTTGAATATCCAGTTTCTCGCCGGGCTTATG	
RAA-R1	TATTTACTCCGTGAAATCCGTATTCTCCTCGT	
RAA-F2	TAAAATTGCCGGTGAATGTTTCTGTGCTTA	
RAA-R2	TATTTACTCCGTGAAATCCGTATTCTCCTC	
RAA-F3	TTGAATATCCAGTTTCTCGCCGGGCTTATG	
RAA-R3	GATATTTACTCCGTGAAATCCGTATTCTCCTC	
RAA-F4	GCTTATGAAATTATTAATCGTAAAAGGGCAAC	
RAA-R4	TAAAACAACTGGAACTCCGATATTTACTCCGT	
ssDNA	FAM-TTATT-BHQ1/FAM-TTATT-Biotin	
Mhp P36 F1	AATTGGTGCTGGAAATGTCGGAA	
Mhp P36 R1	AATTCCGTTTGCCCCTAAAACA	
Mhp GB-F	GAGCCTTCAAGCTTCACCAAGA	Chinese national standard (GB/T 35909-2018)
Mhp GB-R	TGTGTTAGTGACTTTTGCCACC	
Mhp-SN-F	CGGAAATTCCTTCCTTTA	Chinese entry-exit inspection and quarantine industry standard (SN/T4104-2015)
Mhp-SN-R	TCAGGGTTAATATCAATAATTC	
Mhp-SN-probe	FAM-AAGTCCTTGATTCATTGCTGC-TAMRA	

### 2.3 Preparation of standard plasmid

Plasmid was constructed by PCR amplification of genomic DNA using the primer pairs Mhp P36 F1 and R1 (**Table 1**) designed in the conserved regions of the *P36* gene. The PCR products were purified using a gel extraction kit (Omega, Norcross, GA, USA) according to the manufacturer’s instructions. The purified fragments were ligated into the pMD-18T vector (Takara Bio Inc., Dalian, China) and transformed into DH5α competent cells (Takara Bio Inc.) for overnight culture, resulting in plasmid isolation. The concentration of the plasmid was measured using a NanoDrop spectrophotometer (Thermo Fisher Scientific), and the standard plasmid copy number was calculated using the following formula: copies/μL = (A260 (ng/μL) × 10^−9^ × 6.02 × 10^23^)/(DNA length × 650). A ten-fold dilution series ranging from 1 × 10^8^ to 1 × 10^-1^ copies/μL of the standard plasmid was prepared using EASY Dilution (Takara Bio Inc.) and stored at -20°C for subsequent experiments.

### 2.4 RAA amplification and primer design

Standard RAA reactions were conducted according to the instructions of the Basic Nucleic Acid Amplification Kit (ZC Bio-Sci&Tech Co. Ltd, Hangzhou, China). Each reaction mixture contained 25 µL of A Buffer, 2 µL each of forward and reverse primers (10 µM), 5 µL of genomic DNA, 2.5 µL of B buffer, and 13.5 µL of nuclease-free H_2_O. The mixture was incubated in a water bath at 39°C for 30 min. Six primer pairs, denoted as RAA-F1/R1, RAA-F2/R2, RAA-F3/R3, RAA-F4/R3, RAA-F4/R4, and RAA-F4/R2 (**Table 1**), were generated using Primer 5.0 software. These primers were designed based on sequences flanking the crRNA probe region, ensuring no overlap with the crRNA region. The design parameters for these primers include a product length range of 150-250 bp, primer length between 30 and 35 bp, and GC content ranging from 30% to 70%.

### 2.5 Screening of RAA primers and optimization of amplification time

Six primer pairs (RAA-F1/R1, RAA-F2/R2, RAA-F3/R3, RAA-F4/R3, RAA-F4/R4 and RAA-F4/R2) (**Table 1**) were used to perform RAA reactions separately serving standard plasmids of 1 × 10^3^ copies/µL as a template. Primer pairs with higher amplification efficiency and specificity were conceived as candidates. Then, the amplification template was switched from standard plasmid to genome, and following the above procedure, the primer pair with the highest specificity and amplification efficiency among the candidate primer pairs was selected for subsequent experiments. Furthermore, we explored the optimal RAA amplification time. Reaction times of 15 min, 20 min, 25 min and 30 min were tested, with each experiment repeated three times. By comprehensively analyzing the fluorescence generation time, fluorescence curve trends, and fluorescence intensity influenced by the five different RAA reaction times, the optimal RAA reaction time was determined.

### 2.6 Establishment and optimization of the CRISPR/Cas12a reaction system

The unoptimized reaction system (50 μL) consisted of the following mixture: 1 μL LbCas12a (5 µM) (Bio-lifesci Co., Ltd., Guangzhou, China), 5 μL 10× buffer, 2 μL crRNA (5 µM) (*in vitro* synthesis), 2 μL ssDNA reporter probe (10 µM) (Sangon Biotech Co., Ltd., Shanghai, China), 3 μL template, with the remainder made up with nuclease-free H_2_O. The mixture was incubated for 25 min at 37°C in a water bath and a microplate reader was used for detection (PerkinElmer, Waltham, MA, USA). The crRNA was screened using this reaction system and conditions and the most suitable crRNA was identified based on the final fluorescence intensity and reaction efficiency for subsequent experiments. In the assay, the final concentrations of LbCas12a, crRNA and ssDNA were optimized using the checkerboard method to optimize concentrations of LbCas12a and crRNA by fixing other components, which was set up with 30, 60, 120, 180 and 240 nM of crRNA and 20, 40, 60, 80, 100 and 200 nM of LbCas12a. ssDNA was optimized by fixing other components and adjusting the concentration to 100, 200, 300, 400 and 500 nM. Experimental data measured using a microplate reader (PerkinElmer) were processed to determine the optimal concentrations of LbCas12a, crRNA and ssDNA for the final reaction system.

### 2.7 Readout formats

The results can be expressed in terms of fluorescence using a microplate reader and visualized under blue light or through lateral flow assay (LFA). For fluorescence detection, the ssDNA reporter is labeled with FAM and BHQ1 in the fluorescent and quencher groups, respectively. We use a microplate reader (PerkinElmer) with excitation and emission wavelengths set at 494 nm and 518 nm to measure the fluorescence intensity. Additionally, a blue light transilluminator can be used to observe fluorescence, where the presence of green fluorescence serves as an indicator of the target DNA’s existence. For LFA detection, the ssDNA reporter is labeled with FAM and biotin at the 5’ and 3’ ends, respectively. Following the instructions, we place the lateral flow assay strip (GenDx Biotech Co., Ltd., Suzhou, China) into the incubation product, yielding results within 2 min. The lateral flow strip comprises three distinct sections: the sample zone, the control line and the test line. The sample zone is coated with gold-labeled anti-FAM antibodies. The control line area is covered with streptavidin, which captures uncleaved ssDNA reporter molecules labeled with biotin. The test line area is coated with anti-mouse antibodies designed to detect the cleaved ssDNA reporter labeled with gold nanoparticles. For negative samples, the gold-labeled anti-biotin antibodies fully bind to the abundant biotin-labeled ssDNA reporter, resulting in complexes that are intercepted by anti-FAM antibodies at the control line. Conversely, for positive samples, the ssDNA reporter is cleaved, leading to the accumulation of gold-labeled anti-biotin antibody at the test line. The colorimetric process should ideally be completed within 4 min. Positive results are indicated by the presence of a red control line and test line, or only a red test line. In contrast, results are considered negative when only the control line shows a red color, and invalid when no lines are displayed.

### 2.8 Specificity and sensitivity of the RAA-CRISPR/Cas12a fluorescence detection method

In order to investigate the specificity of the RAA-CRISPR/Cas12a fluorescence detection method, genome DNA of *M. hyopneumoniae*, *M. hyorhinis*, *A. pleuropneumoniae*, *H. parasuis*, *S. suis*, *P. multocida*, PCV2, PRV, *M. capricolum*, *M. synoviae* and *M. gallisepticum* and genome cDNA of PRRSV and SIV were detected by the final reaction system. Meanwhile, nuclease-free H_2_O was used as a negative control.

To evaluate the sensitivity of the RAA-CRISPR/Cas12a fluorescence detection method, 10-fold dilutions of standard plasmids ranging from 1 × 10^4^ to 1 × 10^-1^ copies/μL and 5 copies/μL of standard plasmid were used as templates to determine the limit of detection (LoD) for the RAA-CRISPR/Cas12a-fluorescence. Additionally, using the 10-fold dilution of 1 × 10^8^–1 × 10^-1^ copies/μL of the standard plasmid as templates, we tested the sensitivity of qPCR according to the recommended Chinese entry–exit inspection and quarantine industry standard (SN/T4104-2015). The qPCR reaction mixture (20 μL) contained 10 μL Premix ExTaq (probe qPCR) (2×), 0.4 μL of each Mhp-SN-F/R primer (10 µM), 0.8 μL Mhp-SN-probe (10 µM), 0.4 μL Rox Reference DyeII, 5 μL template and 3 μL nuclease-free H_2_O. The cycling conditions were set as follows: initial denaturation at 95°C for 3 min, subsequently proceeding to 40 cycles, each consisting of denaturation at 95°C for 15 seconds and annealing at 55°C for 45 seconds.

### 2.9 Optimization of the RAA-CRISPR/Cas12a LFA time

The reaction time of the RAA-CRISPR/Cas12a lateral flow assay was optimized for use in the final reaction system. Four sets with reaction times of 5 min, 10 min, 15 min and 20 min were optimized using 1 × 10^2^ copies/µL of standard plasmid as templates, and incubated in a 37°C water bath to determine the minimum reaction time. The optimal reaction time was selected by observing the test line and control line.

### 2.10 Specificity and sensitivity of the RAA-CRISPR/Cas12a LFA

In order to evaluate the specificity of the RAA-CRISPR/Cas12a LFA, the thirteen pathogens *M. hyopneumoniae*, *M. hyorhinis*, *A. pleuropneumoniae*, *H. parasuis*, *S. suis*, *P. multocida*, PRRSV, SIV, PCV2, PRV, *M. capricolum*, *M. synoviae* and *M. gallisepticum* were analyzed. Nuclease-free H_2_O was used as a negative control.

For sensitivity assessment, 10-fold serial dilutions of 1 × 10^4^–1 × 10^-1^ copies/μL and 5 copies/μL of the standard plasmid were used as templates to determine the LoD for the RAA-CRISPR/Cas12a LFA.

Specificity and sensitivity of *M. hyopneumoniae* were determined by observing the control and detection lines of the test strips.

### 2.11 Assays of clinical sample

Genomic DNA was extracted from 51 lung tissue samples (17 apical lobes, 17 cardiac lobes and 17 diaphragmatic lobes) using a blood/cell/tissue genomic DNA extraction kit (Tiangen Biotech Co. Ltd., Beijing, China) and from 25 nasal swab samples using a bacterial genomic DNA extraction kit (Tiangen Biotech Co. Ltd.). The DNA samples were tested using the final RAA-CRISPR/Cas12a-fluorescence and RAA-CRISPR/Cas12a LFA. To validate the clinical performance, the same samples were analyzed by PCR recommended by the Chinese national standard (GB/T 35909-2018) and qPCR recommended by the Chinese entry–exit inspection and quarantine industry standard (SN/T4104-2015). Genomic DNA of *M. hyopneumoniae* and nuclease-free H_2_O were used as positive and negative controls, respectively. The reaction mixture (25 μL) for PCR contained 12.5 μL 2× Taq PCR StarMix (Dye), 2 μL of each Mhp-GB-F/R primer (10 µM), 3 μL template and 5.5 μL nuclease-free H_2_O. The reaction conditions were set as follows: pre-denaturation at 94°C for 2 min, followed by PCR performed for 30 cycles of denaturation at 94°C for 30 s, annealing at 60°C for 30 s, and extension at 72°C for 1 min, followed by a final extension at 72°C for 10 min.

### 2.12 Statistical analysis

Data analysis and graphic design were processed by GraphPad Prism v.8.1.2 software. Data in the figures are presented as the mean ± standard deviation. One-way analysis of variance (ANOVA) was used for multigroup comparisons. All comparisons were considered statistically significant at *P* < 0.05 and highly significant at *P* < 0.001.

## 3 Results

### 3.1 Schematic representation of the RAA combined with CRISPR/Cas12a system rapid detection platform

**Figure 1:**
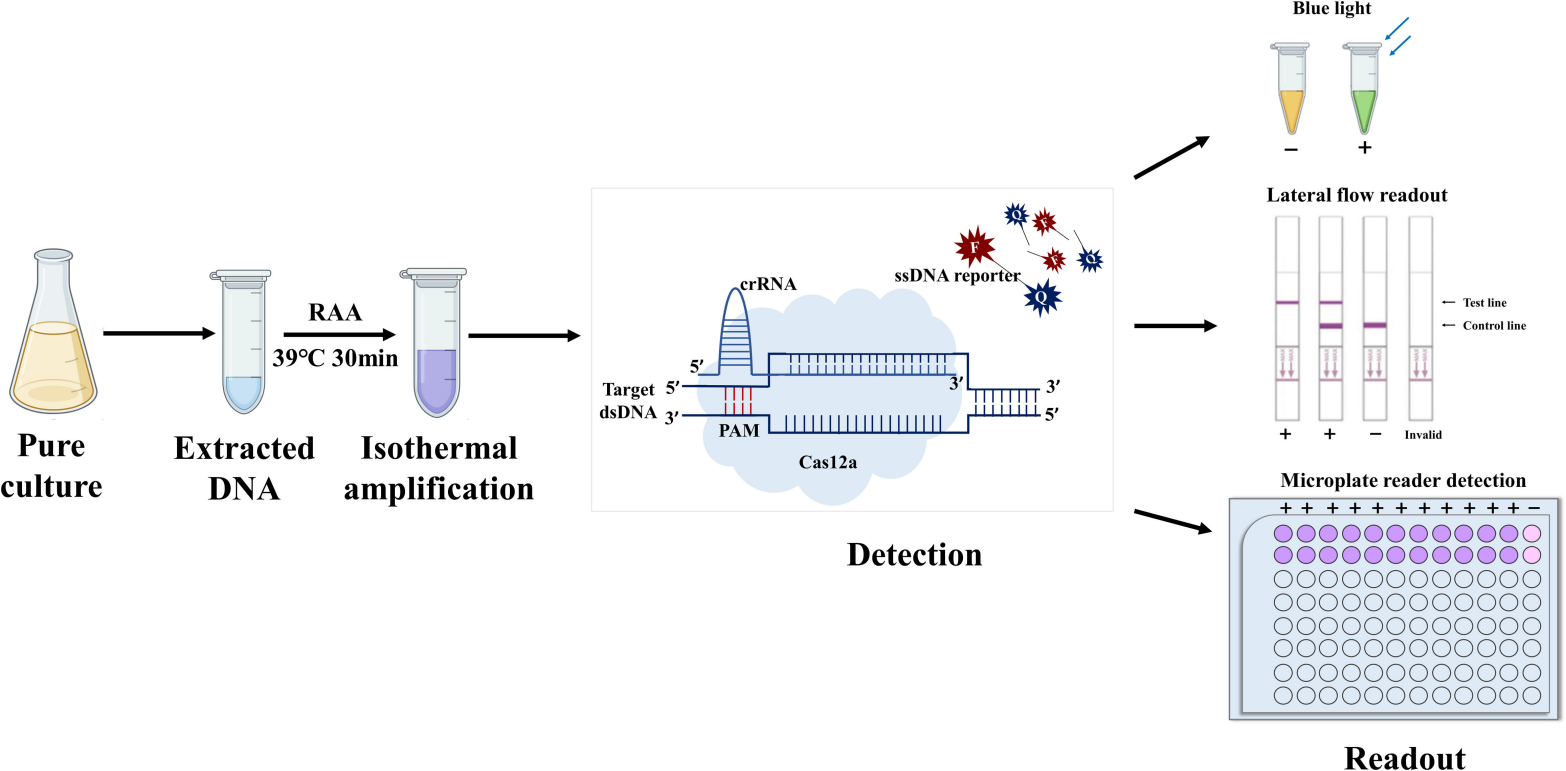
Schematic diagram of the combined RAA–CRISPR/Cas12a system. The procedure comprised four main steps, i) extraction of the genome from pure cultures of bacteria; ii) isothermal amplification; iii) complex formation of Cas12a, crRNA, and isothermal amplification products, as well as activation and cleavage of fluorescently-labeled reporter genes by Cas12a; and iv) readout of results by the naked eye, flowmeter strips, or fluorescence.

An overview of the rapid detection platform for *M. hyopneumoniae* based on RAA-CRISPR/Cas12a is depicted in **Figure 1**. First, genomic DNA is extracted from bacterial culture using a bacterial genomic DNA extraction kit. Next, the target DNA is amplified through RAA at 39°C, followed by transfer of the RAA products into the CRISPR/Cas12a reaction system. Finally, the presence of *M. hyopneumoniae* is determined by visual inspection under blue light, utilization of a lateral flow assay, or fluorescence measured using a microplate reader. The entire process can be completed within one hour.

### 3.2 Screening of RAA primers and optimization of amplification time

**Figure 2:**
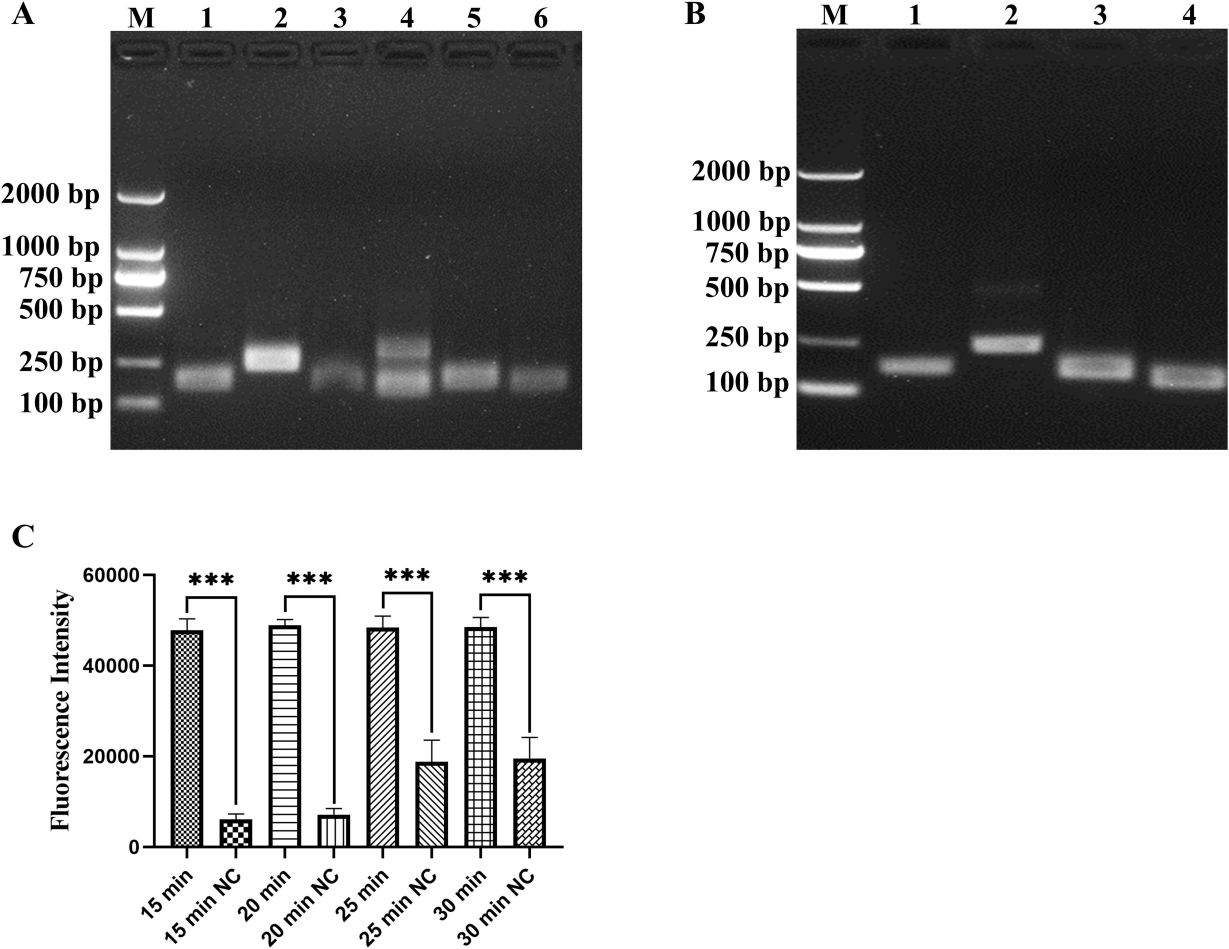
Screening of RAA primers and optimization of amplification time. **(A)** Screening primer pairs based on standard plasmid of 1 × 10^3^ copies/µL, lanes 1–6 represent the primer pairs RAA-F1/R1, RAA-F2/R2, RAA-F3/R3, RAA-F4/R3, RAA-F4/R4 and RAA-F4/R2, respectively. **(B)** Screening primer pairs by DNA of *M. hyopneumoniae*, lanes 1–4 represent the primer pairs RAA-F1/R1, RAA-F2/R2, RAA-F4/R4 and RAA-F4/R2, respectively. **(C)** Optimization of RAA reaction time, tested at 15, 20, 25 and 30 min, with nuclease-free H_2_O as a negative control (NC). ***p < 0.001.

Because the primer sequences are important in RAA amplification, primer screening is necessary. We used standard plasmid and DNA of *M. hyopneumoniae* as templates to identify the optimal primer pair. Four primer pairs (RAA-F1/R1, RAA-F2/R2, RAA-F4/R4, RAA-F4/R2) were selected using a standard plasmid of 1 × 10^3^ copies/µL as a template based on the amplification profile (**Figure 2A**). Then, RAA-F1/R1 was selected for subsequent experiments, as it exhibited the most efficient performance as well as being highly specific when tested using DNA of *M. hyopneumoniae* as a template (**Figure 2B**). Based on the five sets of reaction times designed for the RAA, the optimal RAA reaction time was determined to be 15 min by considering the fluorescence intensity and background fluorescence values influenced by different reaction durations (**Figure 2C**, *P* < 0.001).

### 3.3 Screening crRNA and optimization of the reaction system

**Figure 3:**
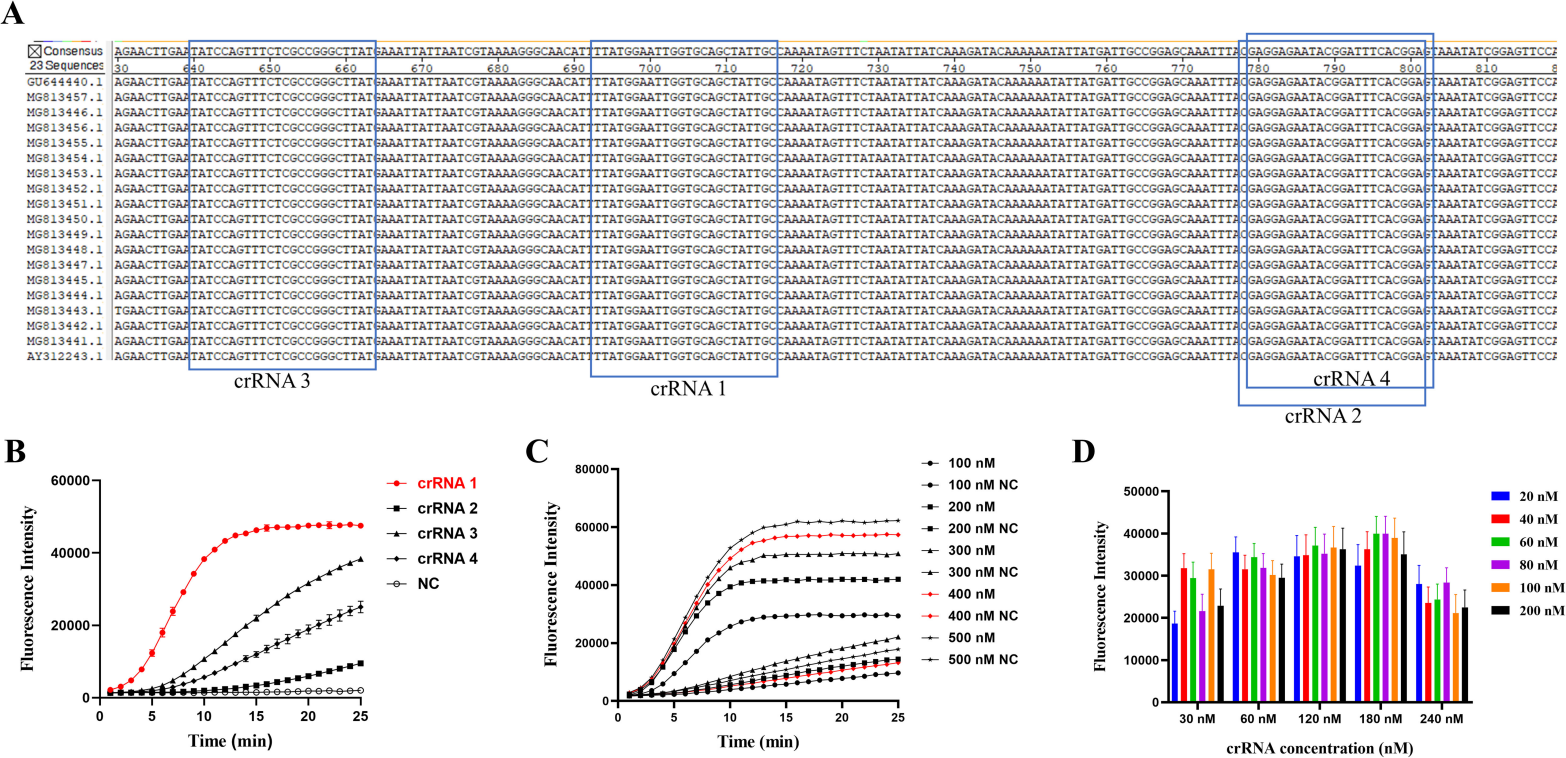
Screening of specific crRNA for the *P36* gene and optimization of the CRISPR/Cas12a reaction system. **(A)** Positional information of the four crRNAs. **(B)** Performance of the four crRNAs was determined by testing the DNA of *M. hyopneumoniae*. **(C)** The optimal fluorescence curves were compared with different concentrations of ssDNA reporter in the CRISPR/Cas12a reaction system. **(D)** Optimization of LbCas12a and crRNA concentrations by the checkerboard method. Endpoint fluorescence readings with LbCas12a (20, 40, 60, 80, 100 and 200 nM) and different concentrations (30, 60, 120, 180 and 240 nM) of crRNA, with nuclease-free H_2_O as a negative control (NC).

Four designed crRNA probes were capable of recognizing distinct 24-nucleotide regions of the *P36* gene (**Figure 3A**). Among them, crRNA 1 exhibited the highest fluorescence value (**Figure 3B**) and the fastest reaction rate. Therefore, crRNA 1 was selected for use in subsequent experiments. The final optimized reaction system comprised 0.6 μL LbCas12a (final concentration of 60 nM) (**Figure 3D**), 5 μL 10 × buffer, 1.8 μL crRNA (final concentration of 180 nM) (**Figure 3D**), 2 μL ssDNA reporter probe (final concentration of 400 nM) (**Figure 3C**), and 3 μL RAA product, with the volume made up to 50 μL with nuclease-free H_2_O.

### 3.4 Specificity and sensitivity of the RAA-CRISPR/Cas12a fluorescence detection system

**Figure 4:**
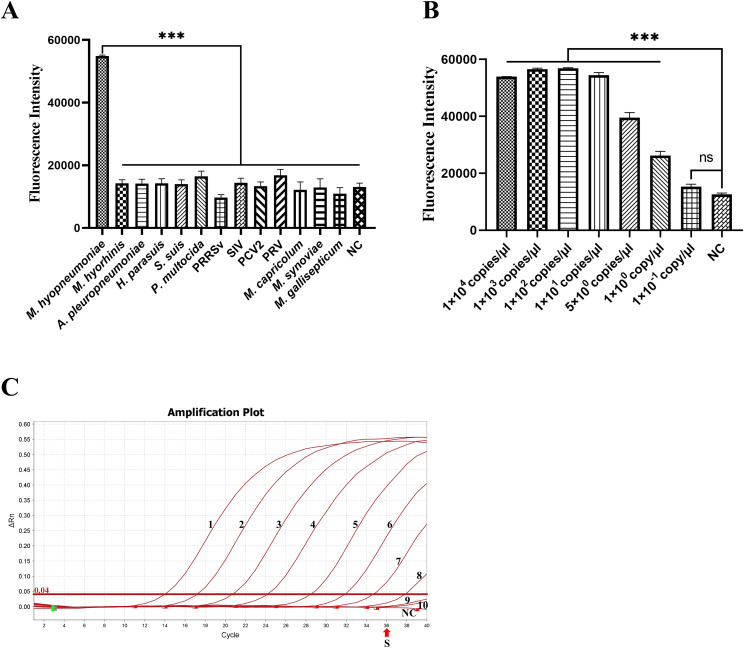
Specificity and sensitivity of the RAA-CRISPR/Cas12a fluorescence for *M. hyopneumoniae*. **(A)** Specificity for thirteen pathogens (*M. hyopneumoniae*, *M. hyorhinis*, *A. pleuropneumoniae*, *H*. *parasuis*, *S. suis*, *P. multocida*, PRRSV, SIV, PCV2, PRV, *M. capricolum*, *M. synoviae* and *M. gallisepticum*) was determined by reading the endpoint fluorescence. **(B)** The sensitivity of the RAA-CRISPR/Cas12a fluorescence for the serially-diluted 10-fold 1 × 10^4^ to 1 × 10^-1^ copies/µL and 5 copies/μL of standard plasmids. **(C)** The sensitivity of qPCR (SN/T4104-2015) for the serially diluted standard plasmid. The numbers 1–10 represent the 1 × 10^8^ to 1 × 10^-1^ copies/µL. The “S” indicates the standard for positive samples, with nuclease-free H_2_O as a negative control (NC). ***p < 0.001.

Genomic DNA of *M. hyopneumoniae*, *M. hyorhinis*, *A. pleuropneumoniae*, *H. parasuis*, *S. suis*, *P. multocida*, PCV2, PRV, *M. capricolum*, *M. synoviae* and *M. gallisepticum* and genomic cDNA of PRRSV and SIV as templates were tested to evaluate the specificity of the RAA-CRISPR/Cas12a-fluorescence system. Only DNA of *M. hyopneumoniae* reacted rapidly with high fluorescence intensity (*P* < 0.001) and did not cross-react with other pathogens (**Figure 4A**). The results showed that the RAA-CRISPR/Cas12a fluorescence detection system was highly specific.

A 10-fold serial dilution series and 5 copies/μL of standard plasmids were used as templates for the RAA-CRISPR/Cas12a-fluorescence. The result showed that the LoD of the fluorescence assay was 1 copy/μL for *M. hyopneumoniae* (**Figure 4B**, *P* < 0.001). At the same, the qPCR recommended by entry–exit inspection and quarantine industry standard (SN/T4104-2015) detected 1 × 10^2^ copies/μL (CT < 36) (**Figure 4C**). This result indicates that the fluorescence assay is highly sensitive, being 100-fold more sensitive than qPCR.

### 3.5 Optimization of the RAA-CRISPR/Cas12a LFA time

**Figure 5:**
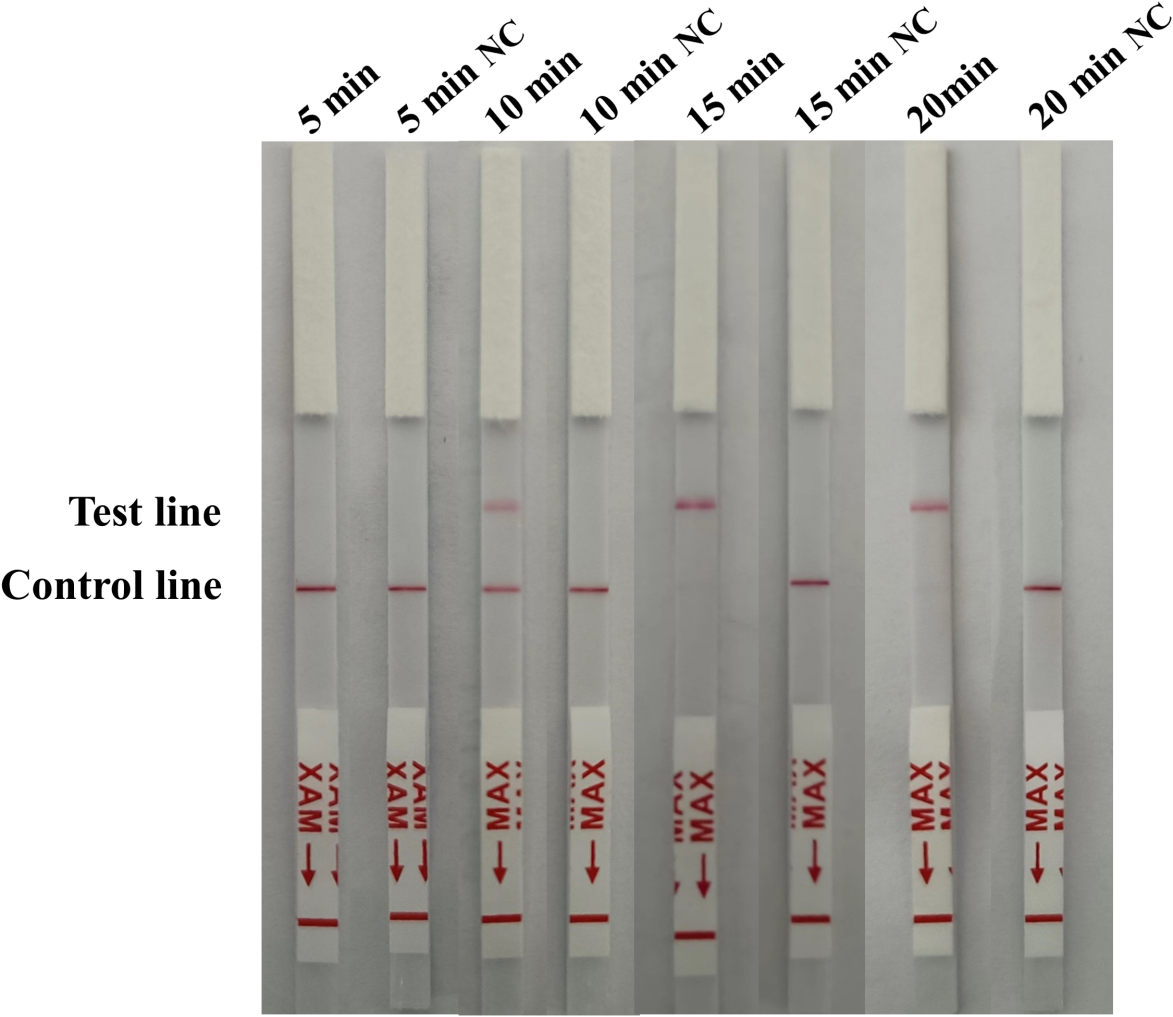
Optimization of the RAA-CRISPR/Cas12a LFA time. For optimization of the reaction time (5, 10, 15 and 20 min) 1 × 10^2^ copies/µL of standard plasmid was used as a template, with nuclease-free H_2_O as a negative control (NC).

The reaction time of the RAA-CRISPR/Cas12a lateral flow assay was optimized based on the final reaction system. When 1 × 10^2^ copies/µL of standard plasmid was used as a template, the lateral flow strip only displayed the detection line after incubation for 15 min and 20 min (**Figure 5**), indicating that it takes a minimum of 15 min for the reaction to be complete. Thus the shortest available reaction time for the RAA-CRISPR/Cas12a LFA was 15 min.

### 3.6 Specificity and sensitivity of the RAA-CRISPR/Cas12a LFA

**Figure 6:**
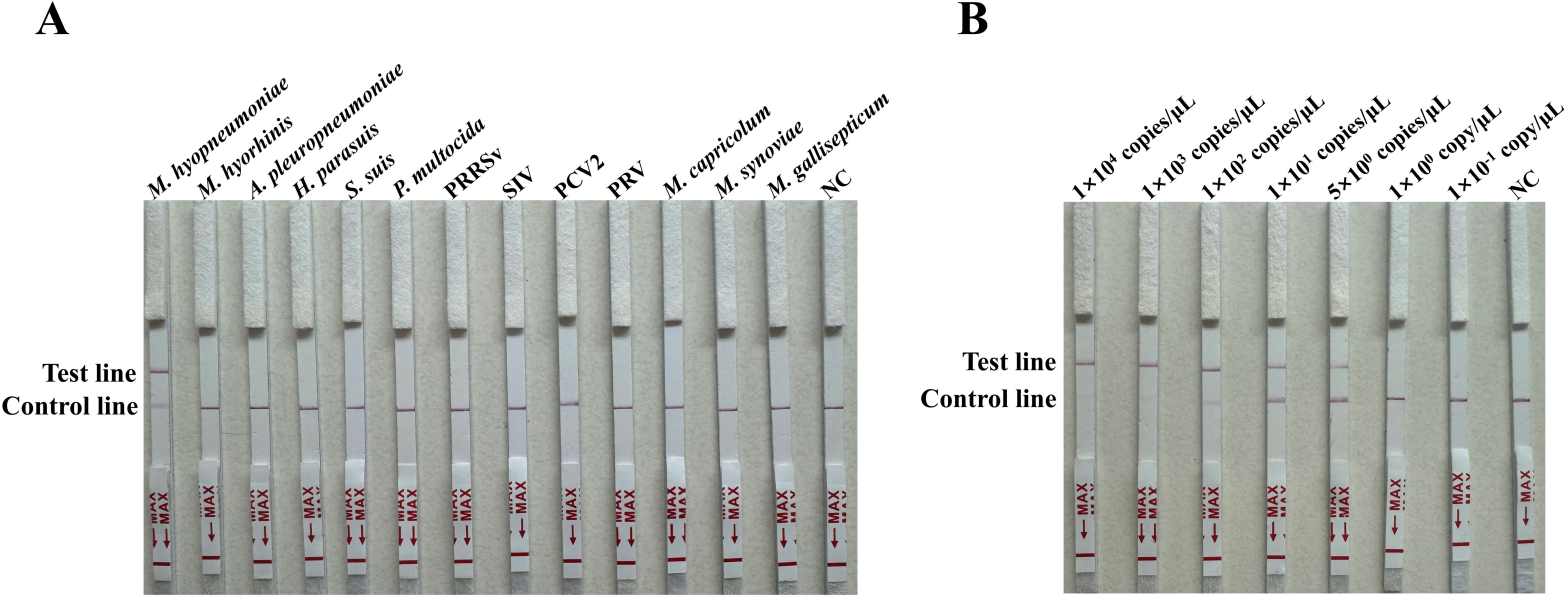
Specificity and sensitivity of the RAA-CRISPR/Cas12a LFA for *M. hyopneumoniae*. **(A)** Specificity for thirteen pathogens (*M. hyopneumoniae*, *M. hyorhinis*, *A. pleuropneumoniae*, *H*. *parasuis*, *S. suis*, *P. multocida*, PRRSV, SIV, PCV2, PRV, *M. capricolum*, *M. synoviae* and *M. gallisepticum*) was determined by observing the development of the red line. **(B)** Sensitivity of the RAA-CRISPR/Cas12a LFA for the serially diluted 10-fold 1 × 10^4^ to 1 × 10^-1^ copies/µL and 5 copies/μL of standard plasmids, with nuclease-free H_2_O as a negative control (NC).

The specificity of the RAA-CRISPR/Cas12a LFA was evaluated for the thirteen pathogens *M. hyopneumoniae*, *M. hyorhinis*, *A. pleuropneumoniae*, *H. parasuis*, *S. suis*, *P. multocida*, PRRSV, SIV, PCV2, PRV, *M. capricolum*, *M. synoviae* and *M. gallisepticum* based on the final reaction system. In the LFA strips the test line was only visible with *M. hyopneumoniae*, for the others only the control line appeared (**Figure 6A**). The sensitivity of the RAA-CRISPR/Cas12a LFA was analyzed using a 10-fold serial dilution and 5 copies/μL of standard plasmids. The LoD was 5 copies/μL (**Figure 6B**).

### 3.7 Application in clinical samples

**Figure 7:**
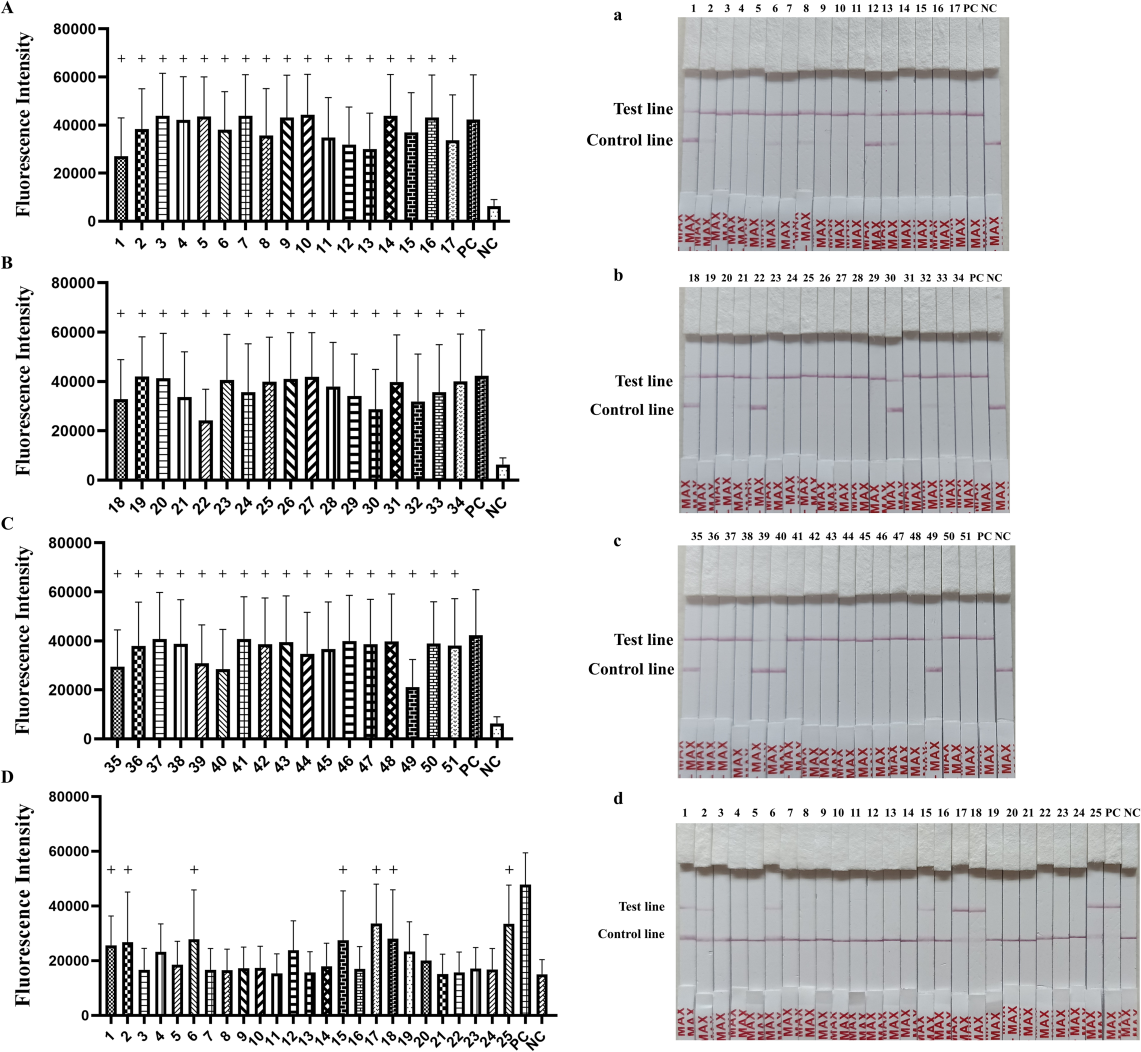
Detection of *M. hyopneumoniae* in clinical samples. Samples from **(A, a)** lung apical lobes, **(B, b)** lung cardiac lobes, **(C, c)** lung diaphragmatic lobes and **(D, d)** nasal swab samples were tested using the RAA-CRISPR/Cas12a detection system. The “+” indicates the positive sample.

In order to evaluate the performance of the established detection platform, 51 lung tissues samples and 25 nasal swab samples were tested using the final RAA-CRISPR/Cas12a fluorescence/LFA (**Figure 7**). The positive rates for 51 lung tissue samples and 25 nasal swab samples were 100% (51/51) and 28% (7/25) in RAA-CRISPR/Cas12a fluorescence (**Table 2**, **Figure 7**, *P* < 0.05). The same results were obtained by RAA-CRISPR/Cas12a LFA (**Table 2**, **Figure 7**). To verify the accuracy of the method, we tested the clinical samples using PCR as recommended by the Chinese national standard (GB/T 35909-2018) and qPCR as recommended by the Chinese entry–exit inspection and quarantine industry standard (SN/T4104-2015). The PCR results indicated a positive rate of 96% (49/51) among 51 lung tissue samples, and the nasal swab samples exhibited a 0% (0/25) positive rate among 25 samples (**Table 2**). Additionally, the 51 lung tissue samples tested by qPCR were 100% (51/51) positive, meanwhile, the nasal swab samples showed a 16% (4/25) positive rate among the 25 samples examined (**Table 2**). The results obtained using the RAA-CRISPR/Cas12a fluorescence/LFA method were consistent with those obtained from PCR (GB/T 35909-2018) and qPCR (SN/T4104-2015). This suggests that the novel approach is not only accurate and reliable but also exhibits a higher sensitivity.

**Table 2:** Detection of clinical samples by the RAA-CRISPR/Cas12a system, qPCR and PCR methods.

Methods	lung tissue samples			nasal swab samples		
	Number	Positive/Total Number	Positive rate	Number	Positive/Total Number	Positive rate
RAA-CRISPR/Cas12a- fluorescence	51	51/51	100%	25	7/25	28%
RAA-CRISPR/Cas12a- LFA		51/51	100%		7/25	28%
qPCR (SN/T4104-2015)		51/51 (CT=20.95-35.65)	100%		4/25 (CT=33.89-34.97)	16%
PCR (GB/T 35909-2018)		49/51	96%		0/25	0%

## 4 Discussion

*M. hyopneumoniae* has a significant economic impact on the swine industry (Maes et al., 2021). *M. hyopneumoniae* infection is widespread globally and prone to facilitating concurrent infections among animals, particularly with other porcine respiratory pathogens (Maes et al., 2020). Currently, the molecular diagnostic techniques of PCR, nPCR and qPCR are well established in the literature (Cai et al., 2007; Calsamiglia et al., 1999; Canturri et al., 2024; Chen et al., 2018; Dubosson et al., 2004; Moiso et al., 2020; Strait et al., 2008) and in the Chinese national standard (GB/T 35909-2018), the Chinese agricultural industry standard (NY/T 1186-2017) and the Chinese entry–exit inspection and quarantine industry standard (SN/T4104-2015). Although these techniques have been widely validated as useful tools for detecting the disease, they are still not convenient for use in frontline sites due to the requirements for expensive instruments and specialized operating systems. Therefore, we envisaged an urgent need for a method for detection of *M. hyopneumoniae* based on the combination of RAA and CRISPR/Cas12a which required minimal equipment, and was highly sensitive and capable of rapid detection.

RAA, a novel isothermal nucleic acid amplification technique, enables rapid amplification of DNA or RNA within 30 min at relatively low temperatures (37–42°C) using a simple water bath (Li et al., 2023). This technology relies on three fundamental enzymes: recombinase, single-stranded DNA-binding protein (SSB) and strand-displacing DNA polymerase (Li et al., 2020; Tu et al., 2021), which replace the denaturation cycles required in conventional PCR. The CRISPR/Cas system, known for its reliability, sensitivity, and specificity, has emerged as a valuable tool for genomic editing and nucleic acid diagnosis (Fapohunda et al., 2022), but the CRISPR/Cas12a system is more suitable for the detection of bacteria. Combining RAA with the CRISPR/Cas12a system (Chen et al., 2018; Yao et al., 2018), when the target nucleic acid is present, the specific product is amplified by RAA and specifically recognized by Cas12a protein and crRNA complex, and at the same time, the Cas12a trans-cutting activity is activated, which cuts the fluorescent reporter probes and emits fluorescence to judge the detection results (Chen et al., 2018). Moreover, a cost analysis comparison of PCR, qPCR, and CRISPR/Cas12a has been reported (Wang et al., 2024), which revealed that the CRISPR/Cas12a system is the easiest to operate and the least demanding in terms of instrumentation, while also allowing for high-throughput assays, both in the laboratory and in on-farm settings.

In our study, we confirmed that RAA-F1/R1 and crRNA 1 serve as the most suitable primer pair and crRNA in the conserved *M. hyopneumoniae P36* gene. For RAA we performed the amplification at 37°C for 15 min, which is 15 min shorter than the time given in the initial instructions. We presented the RAA-CRISPR/Cas12a system results in two forms, either measuring fluorescence values with a microplate reader or using lateral flow test strips. The RAA-CRISPR/Cas12a fluorescence and RAA-CRISPR/Cas12a LFA both proved to be highly specific for *M. hyopneumoniae* and showed no cross-reactivity with other pathogens (*M. hyorhinis*, *A. pleuropneumoniae*, *H. parasuis*, *S. suis*, *P. multocida*, PRRSV, SIV, PCV2, PRV, *M. capricolum*, *M. synoviae* and *M. gallisepticum*). The sensitivity test revealed a LoD of 1 copy/μL and 5 copies/μL of the RAA-CRISPR/Cas12a-fluorescence assay and RAA-CRISPR/Cas12a- LFA, respectively. Both of these methods can serve as the qualitative test for samples, rather than quantitative analysis. Furthermore, we tested 51 lung tissue samples and 25 nasal swab samples with the RAA-CRISPR/Cas12a system to verify its practicality and usefulness in clinical samples. Fifty-one (100%) positive lung tissue samples and seven (28%) positive nasal swab samples were detected by the RAA-CRISPR/Cas12a fluorescence and RAA-CRISPR/Cas12a LFA, respectively. It can be seen that *M. hyopneumoniae* infection in pigs should not be ignored and should be detected in time to prevent the spread of the infection. Meanwhile, we also tested the 51 lung tissue samples and 25 nasal swab samples by the PCR method recommended by the Chinese national standard (GB/T35909-2018) and by the qPCR method recommended by the Chinese entry–exit inspection and quarantine industry standard (SN/T4104-2015) to verify the accuracy of the RAA-CRISPR/Cas12a system. Forty-nine (96%) of the 51 lung tissue samples and 0 (0%) of the 25 nasal swab samples were detected by PCR. The qPCR testing revealed that 51 (100%) of the 51 lung tissue samples were positive, whereas 4 (16%) of the 25 nasal swab samples tested positive. The results showed consistency rates of 100% between the three methods. Above all, compared with PCR and qPCR, the RAA-CRISPR/Cas12a system is not only more sensitive, but also significantly more stable.

## 5 Conclusion

In conclusion, RAA-CRISPR/Cas12a was established and used as a rapid, highly specific, cost-effective diagnostic and portable detection method for *M. hyopneumoniae*. The method can be used in a high-throughput mode in swine fields or microbiology diagnostic laboratories, with results obtained in less than one hour. It provides an effective means for field diagnosis of *M. hyopneumoniae* and microbiological quality control of experimental swine.

## Data Availability

The original contributions presented in the study are included in the article/supplementary material. Further inquiries can be directed to the corresponding authors.

## References

[B1] Assavacheep P. Thanawongnuwech R. (2022). Porcine respiratory disease complex: dynamics of polymicrobial infections and management strategies after the introduction of the african swine fever. Front. Vet. Sci. 9. doi: 10.3389/fvets.2022.1048861 PMC9732666 36504860

[B2] Cai H. Y. van Dreumel T. McEwen B. Hornby G. Bell-Rogers P. McRaild P.. (2007). Application and field validation of a pcr assay for the detection of mycoplasma hyopneumoniae from swine lung tissue samples. J. Vet. Diagn. Invest. 19, 91–95. doi: 10.1177/104063870701900115 17459839

[B3] Calsamiglia M. Pijoan C. Trigo A. (1999). Application of a nested polymerase chain reaction assay to detect mycoplasma hyopneumoniae from nasal swabs. J. Vet. Diagn. Invest. 11, 246–251. doi: 10.1177/104063879901100307 10353356

[B4] Canturri A. Galina-Pantoja L. Vonnahme K. Pieters M. (2024). Detection of mycoplasma hyopneumoniae viability using a pcr-based assay. Vet. Microbiol. 292, 110058. doi: 10.1016/j.vetmic.2024.110058 38537399

[B5] Caron J. Ouardani M. Dea S. (2000). Diagnosis and differentiation of mycoplasma hyopneumoniae and mycoplasma hyorhinis infections in pigs by pcr amplification of the p36 and p46 genes. J. Clin. Microbiol. 38, 1390–1396. doi: 10.1128/JCM.38.4.1390-1396.2000 10747113 PMC86451

[B6] Chen J. S. Ma E. Harrington L. B. Da Costa M. Tian X. Palefsky J. M.. (2018). Crispr-cas12a target binding unleashes indiscriminate single-stranded dnase activity. Science. 360, 436–439. doi: 10.1126/science.aar6245 29449511 PMC6628903

[B7] Ding H. Wen Y. Xu Z. Zhou B. Tlili C. Tian Y.. (2021). Development of an elisa for distinguishing convalescent sera with mycoplasma hyopneumoniae infection from hyperimmune sera responses to bacterin vaccination in pigs. Vet. Med. Sci. 7, 1831–1840. doi: 10.1002/vms3.539 34021737 PMC8464267

[B8] Dubosson C. R. Conzelmann C. Miserez R. Boerlin P. Frey J. Zimmermann W.. (2004). Development of two real-time pcr assays for the detection of mycoplasma hyopneumoniae in clinical samples. Vet. Microbiol. 102, 55–65. doi: 10.1016/j.vetmic.2004.05.007 15288927

[B9] Fapohunda F. O. Qiao S. Pan Y. Wang H. Liu Y. Chen Q.. (2022). Crispr cas system: a strategic approach in detection of nucleic acids. Microbiol. Res. 259, 127000. doi: 10.1016/j.micres.2022.127000 35338974

[B10] Feng Z. Bai Y. Yao J. Pharr G. T. Wan X. Xiao S.. (2014). Use of serological and mucosal immune responses to mycoplasma hyopneumoniae antigens p97r1, p46 and p36 in the diagnosis of infection. Veterinary J. 202, 128–133. doi: 10.1016/j.tvjl.2014.06.019 25066030

[B11] Garza-Moreno L. Vilalta C. Pieters M. (2022). Environmental detection of mycoplasma hyopneumoniae in breed-to-wean farms. Res. Vet. Sci. 145, 188–192. doi: 10.1016/j.rvsc.2022.02.009 35231720

[B12] Hao J. Xie L. Yang T. Huo Z. Liu G. Liu Y.. (2023). Naked-eye on-site detection platform for pasteurella multocida based on the crispr-cas12a system coupled with recombinase polymerase amplification. Talanta. 255, 124220. doi: 10.1016/j.talanta.2022.124220 36621165

[B13] Kellner M. J. Koob J. G. Gootenberg J. S. Abudayyeh O. O. Zhang F. (2019). Sherlock: nucleic acid detection with crispr nucleases. Nat. Protoc. 14, 2986–3012. doi: 10.1038/s41596-019-0210-2 31548639 PMC6956564

[B14] Li S. Cheng Q. Liu J. Nie X. Zhao G. Wang J. (2018a). Crispr-cas12a has both cis- and trans-cleavage activities on single-stranded dna. Cell Res. 28, 491–493. doi: 10.1038/s41422-018 29531313 PMC5939048

[B15] Li S. Cheng Q. Wang J. Li X. Zhang Z. Gao S.. (2018b). Crispr-cas12a-assisted nucleic acid detection. Cell Discovery 4, 20. doi: 10.1038/s41421-018-0028-z 29707234 PMC5913299

[B16] Li J. Minion F. C. Petersen A. C. Jiang F. Yang S. Guo P.. (2013). Loop-mediated isothermal amplification for rapid and convenient detection of mycoplasma hyopneumoniae. World J. Microbiol. Biotechnol. 29, 607–616. doi: 10.1007/s11274-012-1216-x 23184577

[B17] Li Y. Yu Z. Jiao S. Liu Y. Ni H. Wang Y. (2020). Development of a recombinase-aided amplification assay for rapid and sensitive detection of porcine circovirus 3. J. Virol. Methods 282, 113904. doi: 10.1016/j.jviromet.2020.113904 32470487

[B18] Li X. Zhu S. Zhang X. Ren Y. He J. Zhou J.. (2023). Advances in the application of recombinase-aided amplification combined with crispr-cas technology in quick detection of pathogenic microbes. Front. Bioeng. Biotechnol. 11. doi: 10.3389/fbioe.2023.1215466 PMC10502170 37720320

[B19] Liu M. J. Du G. M. Bai F. F. Wu Y. Z. Xiong Q. Y. Feng Z. X.. (2015). A rapid and sensitive loop-mediated isothermal amplification procedure (lamp) for mycoplasma hyopneumoniae detection based on the p36 gene. Genet. Mol. Res. 14, 4677–4686. doi: 10.4238/2015.May.4.27 25966242

[B20] Liu M. DU G. Zhang Y. Wu Y. Wang H. Li B.. (2016). Development of a blocking elisa for detection of mycoplasma hyopneumoniae infection based on a monoclonal antibody against protein p65. J. Vet. Med. Sci. 78, 1319–1322. doi: 10.1292/jvms.15-0438 27075114 PMC5053934

[B21] Liu L. Li R. Zhang R. Wang J. An Q. Han Q.. (2019). Rapid and sensitive detection of mycoplasma hyopneumoniae by recombinase polymerase amplification assay. J. Microbiol. Methods 159, 56–61. doi: 10.1016/j.mimet.2019.02.015 30807776

[B22] Luan T. Wang L. Zhao J. Luan H. Zhang Y. Wang C.. (2022). A crispr/cas12a-assisted rapid detection platform by biosensing the apxiva of actinobacillus pleuropneumoniae. Front. Microbiol. 13. doi: 10.3389/fmicb.2022.928307 PMC9493679 36160205

[B23] Maes D. Boyen F. Devriendt B. Kuhnert P. Summerfield A. Haesebrouck F. (2021). Perspectives for improvement of mycoplasma hyopneumoniae vaccines in pigs. Vet. Res. 52, 67. doi: 10.1186/s13567-021-00941-x 33964969 PMC8106180

[B24] Maes D. Boyen F. Haesebrouck F. Gautier-Bouchardon A. V. (2020). Antimicrobial treatment of mycoplasma hyopneumoniae infections. Veterinary J. 259-260, 105474. doi: 10.1016/j.tvjl.2020.105474 32553237

[B25] Maes D. Segales J. Meyns T. Sibila M. Pieters M. Haesebrouck F. (2008). Control of mycoplasma hyopneumoniae infections in pigs. Vet. Microbiol. 126, 297–309. doi: 10.1016/j.vetmic.2007.09.008 17964089 PMC7130725

[B26] Maes D. Sibila M. Kuhnert P. Segalés J. Haesebrouck F. Pieters M. (2018). Update on mycoplasma hyopneumoniae infections in pigs: knowledge gaps for improved disease control. Transbound Emerg. Dis. 65, 110–124. doi: 10.1111/tbed.12677 28834294

[B27] Moiso N. Pieters M. Degano F. Vissio C. Camacho P. Estanguet A.. (2020). Detection of mycoplasma hyopneumoniae in nasal and laryngeal swab specimens in endemically infected pig herds. Vet. Rec. 186, 27. doi: 10.1136/vr.105525 31732508

[B28] Montague T. G. Cruz J. M. Gagnon J. A. Church G. M. Valen E. (2014). Chopchop: a crispr/cas9 and talen web tool for genome editing. Nucleic. Acids Res. 42, W401–W407. doi: 10.1093/nar/gku410 24861617 PMC4086086

[B29] Sibila M. Pieters M. Molitor T. Maes D. Haesebrouck F. Segalés J. (2009). Current perspectives on the diagnosis and epidemiology of mycoplasma hyopneumoniae infection. Veterinary J. 181, 221–231. doi: 10.1016/j.tvjl.2008.02.020 PMC7110805 18396428

[B30] Strait E. L. Madsen M. L. Minion F. C. Christopher-Hennings J. Dammen M. Jones K. R.. (2008). Real-time pcr assays to address genetic diversity among strains of mycoplasma hyopneumoniae. J. Clin. Microbiol. 46, 2491–2498. doi: 10.1128/JCM.02366-07 18524960 PMC2519509

[B31] Thacker E. L. (2004). Diagnosis of mycoplasma hyopneumoniae. Anim. Health Res. Rev. 5, 317–320. doi: 10.1079/AHR200491 15984347

[B32] Tu F. Yang X. Xu S. Chen D. Zhou L. Ge X.. (2021). Development of a fluorescent probe-based real-time reverse transcription recombinase-aided amplification assay for the rapid detection of classical swine fever virus. Transbound Emerg. Dis. 68, 2017–2027. doi: 10.1111/tbed.13849 32979245

[B33] van Dongen J. E. Berendsen J. T. W. Steenbergen R. D. M. Wolthuis R. M. F. Eijkel J. C. T. Segerink L. I. (2020). Point-of-care crispr/cas nucleic acid detection: recent advances, challenges and opportunities. Biosensors Bioelectronics. 166, 112445. doi: 10.1016/j.bios.2020.112445 32758911 PMC7382963

[B34] Wang L. Sun J. Zhao J. Bai J. Zhang Y. Zhu Y.. (2024). A crispr-cas12a-based platform facilitates the detection and serotyping of streptococcus suis serotype 2. Talanta. 267, 125202. doi: 10.1016/j.talanta.2023.125202 37734291

[B35] Yao R. Liu D. Jia X. Zheng Y. Liu W. Xiao Y. (2018). Crispr-cas9/cas12a biotechnology and application in bacteria. Synth. Syst. Biotechnol. 3, 135–149. doi: 10.1016/j.synbio.2018.09.004 30345399 PMC6190536

[B36] Yuan B. Yuan C. Li L. Long M. Chen Z. (2022). Application of the crispr/cas system in pathogen detection: a review. Molecules. 27, 6999. doi: 10.3390/molecules27206999 36296588 PMC9610700

[B37] Zhang K. Sun Z. Shi K. Yang D. Bian Z. Li Y.. (2023). Rpa-crispr/cas12a-based detection of haemophilus parasuis. Animals. 13, 3317. doi: 10.3390/ani13213317 37958075 PMC10648042

[B38] Zhang H. Wang Y. Gao L. Wang Y. Wei R. (2021). Genotype diversity of mycoplasma hyopneumoniae in chinese swine herds based on multilocus sequence typing. BMC Vet. Res. 17, 347. doi: 10.1186/s12917-021-03059-6 34749727 PMC8574025

[B39] Zimmer F. M. A. L. Paes J. A. Zaha A. Ferreira H. B. (2020). Pathogenicity & virulence of mycoplasma hyopneumoniae. Virulence. 11, 1600–1622. doi: 10.1080/21505594.2020.1842659 33289597 PMC7733983

